# Hidden concerns of sharing research data by low/middle-income country scientists

**DOI:** 10.1080/11287462.2018.1441780

**Published:** 2018-02-26

**Authors:** Louise Bezuidenhout, Ereck Chakauya

**Affiliations:** ^a^ Institute for Science, Innovation and Society, University of Oxford, Oxford, UK; ^b^ Steve Biko Centre for Bioethics, University of the Witwatersrand, Johannesburg, South Africa; ^c^ NEPAD-SANBio (Southern African Network of Biosciences), Pretoria, South Africa

**Keywords:** Open Data, data sharing, life sciences, low/middle-income countries, NEPAD-SANBio

## Abstract

There has considerable interest in bringing low/middle-income countries (LMIC) scientists into discussions on Open Data – both as contributors and users. The establishment of *in situ* data sharing practices within LMIC research institutions is vital for the development of an Open Data landscape in the Global South. Nonetheless, many LMICs have significant challenges – resource provision, research support and extra-laboratory infrastructures. These low-resourced environments shape data sharing activities, but are rarely examined within Open Data discourse. In particular, little attention is given to how these research environments shape scientists’ perceptions of data sharing (dis)incentives. This paper expands on these issues of incentivizing data sharing, using data from a quantitative survey disseminated to life scientists in 13 countries in sub-Saharan Africa. This interrogated not only perceptions of data sharing amongst LMIC scientists, but also how these are connected to the research environments and daily challenges experienced by them. The paper offers a series of analysis around commonly cited (dis)incentives such as data sharing as a means of improving research visibility; sharing and funding; and online connectivity. It identifies key areas that the Open Data community need to consider if true openness in research is to be established in the Global South.

## Introduction

In the last decade, the amount of scientific research occurring in low/middle-income countries (LMICs) has increased considerably. Changes in collaboration and funding structures,^1^ together with improved national support for research agendas (AU-NEPAD, 2010; NEPAD, 2014), have been highly influential in shaping this changing landscape. In addition, increased focus on Open Access to online materials, research and publication support and dedicated networking funding^2^ have all contributed important research resources to these regions.

In response to the increased amounts of scientific data produced in these regions, there has considerable interest in bringing LMIC scientists into discussions on Open Data – both as contributors and users. Indeed, recent projects such as the Africa Open Science Platform^3^ stand as evidence of this commitment. They illustrate the desired future for research in LMICs, whereby they are able to meaningfully be part of the emerging Open Science milieu. Nonetheless, integrating openness – particularly in terms of data – within many LMIC research systems is challenging. Many of these countries have been largely absent from early discussions on openness, and considerable amounts of the emerging discussions (and policies) make little explicit reference to research originating in these low-resourced research settings.

The recognition that research environments in many LMICs differ markedly from high-income countries (HICs) in terms of resource provision, research support and extra-laboratory infrastructures (power, Internet and so forth) complicates immediate integration. Recent empirical research in sub-Saharan Africa (Bezuidenhout, Kelly, Leonelli, & Rappert, 2017; Bezuidenhout, Leonelli, Kelly, & Rappert, 2017; Harle, 2011) have identified a wide range of physical, social and economic challenges that shape LMIC researchers’ ability to engage with data online – both as users and as disseminators. These studies highlight the range of considerations that need to be taken into account in order to develop, situate and perpetuate data sharing activities in LMICs.

Recognizing these differences suggests that many of the lessons learnt by the Open Data community may not be readily transferrable to the Global South. This is particularly true if research environments in LMICs are not to be reduced to a series of HIC-comparisons, namely online/offline, visible/invisible, or funded/unfunded. If one recognises the highly complex and varied environments of LMIC research settings and the challenge that they pose to daily Open Data activities, the need for more nuanced solutions is obvious. In particular, they draw attention to the potential differences involved in incentivizing data sharing amongst LMIC scientists. This paper expands on these issues of incentivizing data sharing, using data from a quantitative survey disseminated to life scientists in 13 countries in sub-Saharan Africa.

## The Open Data movement and modern science

Open Data movement has increasingly become a central element of modern science. It champions the freedom to use, re-use and redistribute data without restrictions beyond a requirement for attribution and share-alike (Molloy, 2011). The Open Data movement is premised on a commitment to justice and beneficence, highlighting a number of key ethical considerations. First, that the increased availability of research data, together with the ever-improving modes of re-analysis, offers considerable opportunities for contributing to future human well-being. Second, that the investment of public funds in the production of research data warrants that the outputs of these studies be readily accessible for re-use and scrutiny (International Council for Science, InterAcademy Partnership, International Social Science Council, & World Academy of Science, 2015).

These ethical considerations are accompanied by recognized epistemic benefits. Heightened transparency and the improved facilitation of self-correction within research offer an important counter to the “replicability crisis” that has rocked science in recent years (Schooler, 2014). Enabling scientists to scrutinize the data contributing to academic publications is widely recognized to be an important means of identifying problematic datasets and/or research programmes.

The recognition of the benefits of increased openness in research has attracted attention from academia, industry and governments around the world (Jin, Wah, Cheng, & Wang, 2015). Statements such as the recent international accord *Open Data in a Big Data World* makes mention of an “Open Data imperative”, where the increased data and idea transmission through the “networked interaction of many minds” (International Council for Science et al., 2015, p. 1) is vital for the future of science. This notion of a data sharing “imperative” is embraced by a rising number of funders and institutions. Consequentially, data sharing is becoming a criterion of research support. Agencies such as the Wellcome Trust have outlined clear policies on data management and sharing that serve as expectations of their grant holders.^4^

Despite the widespread support for the principles of Open Data, the practicalities of sharing research data are recognized to be complicated. Indeed, most data sharing statements recognize that there is no “one size fits all” when it comes to *in situ* activities. The disciplinary community norms, ranges of data types, complicated standards and issues of interoperability, ethical issues and those relating to ownership and intellectual property all play a role in the translation of an ideal of openness into practice. Ultimately, while scientists should aspire to make sure that their data are “FAIR” (free, accessible, interoperable and re-usable) (Wilkinson et al., 2016), how they go about this depends largely on their individual practices and institutional support.

### Incentivizing data sharing

Sharing research data thus involves a commitment from individual scientists. Indeed, the Open Data movement in its current form cannot move forward without the buy-in from individual scientists and their establishment of *in situ* data management and dissemination protocols. In recognition of this, there has been an increasing amount of interest in scoping out the incentives and disincentives that scientists identify as associated with data sharing.

In identifying incentives, the Open Data community often draws on the successes of the Open Access movement. It has been noted that Open Access publications receive more citations than those behind paywalls (Eysenbach, 2006; MacCallum & Parthasarathy, 2006) and similarly, sharing research data is promoted as a way of increasing downstream collaborations. The rise of so-called altmetric pathways^5^ of sharing – through initiatives such as Figshare, professional networking sites and personal web pages – has been shown to be efficacious in increasing the visibility of individual researchers and their work online (Neylon, Wu, Reichelt, Bettencourt, & Chute, 2009; Peters, Kraker, Lex, Gumpenberger, & Gorraiz, 2015). Indeed, data released via these channels have the opportunity to be discussed, annotated, recommended, refuted, commented, read and taught long before it ever appears in the formal citation registry (Priem et al., 2012).

Nonetheless, while the benefits of increased openness in research are widely acknowledged – both within the scientific community as well as with stakeholders – the transition from more traditional research practices to this new paradigm has raised concerns amongst researchers. The loss of intellectual property rights, the misuse of data, or losing out on credit attribution remain key issues that constrain scientists’ involvement in data sharing practices online. In 2014, the publishing house, Wiley, conducted a survey of 2250 scientists in the U.S.A., U.K., Japan, China, Brazil, Australia and Germany that clearly demonstrated these concerns (Ferguson, 2014). While the respondents recognized the importance of sharing data (“it increases the impact and visibility of my research”), common reasons for not sharing centred on the fear of the unknown and loss of control. These included intellectual and property issues, being scooped and misinterpretation or misuse of data (Ferguson, 2014). Saliently, only half of the participants reported engaging in data sharing activities – with the distribution of data sharing varying considerably between countries.

### Data sharing and LMICs

Data practices – while motivated by the ideals discussed above – are thus be best understood as highly varied according to the scientist, data and research community (Borgman, 2012; Tenopir et al., 2011). Interestingly, the majority of discussions on this heterogeneity of practice focus on the variations necessary due to the *characteristics of the data* in question. Far less discussion examines the heterogeneity of practice arising from variations in the *research environments* in which research occurs. In particular, discussions about the physical research environments – the provision of ICTs (information and communication technologies), the design of the work area, technical support, maintenance and so forth – are rarely discussed, or subsumed into discussions on regulation and policy.

It is likely that this emphasis on variable *data* over variable *research environments* is linked to the origins of Open Data discussions in HICs. Issues such as critical resource shortages, the absence of research networks and lack of infrastructural support are often null issues within most HIC research facilities, and are thus excluded from mainstream discussion. As Open Data discussions evolved, this ultimately led to a tendency to “black box” the settings in which researchers generate and share their data. In effect, many discussions assume that a certain “minimum level of resource and service provision” (power, Internet, technical support and so forth) exists throughout the Open Data community that will support the evolution of data sharing practices.

Such assumptions are, of course, not useful when extending Open Data discussions to include LMICs. Qualitative research within LMICs has already identified a range of physical, social and regulatory issues that influence data sharing activities in these settings. These included everything from a lack of research equipment and funds for consumables, to high teaching loads and a reliance on postgraduate students to generate data (Bezuidenhout, Kelly, et al., 2017; Bezuidenhout, Leonelli, et al., 2017). Moreover, these studies clearly represent the complexity of the ICT environments in which these researchers exist. While binaries such as offline/online may continue to exist in some LMIC research institutions, an ever-rising majority have some form of Internet access. In addition to the problems of slower connection speeds, these studies identified a host of other ICT issues – including out-of-date hard/software, computer sharing and time to work online, lack of proxy servers and an inability to access library resources off campus, and a paucity of qualified technical support (Bezuidenhout, Kelly, et al., 2017; Bezuidenhout, Leonelli, et al., 2017; Harle, 2011). Such issues necessarily influence scientists’ enthusiasm for embarking on data sharing activities – as online contributors as well as users.

As the inclusion of LMICs in Open Data discussions continues to be relatively new (Schwegmann, 2013), targeted initiatives to address these challenges are still emerging. Many of these initiatives address recognised ICT limitations in LMICs, such as low processing power for data analysis, curtailed access to online environments, and challenges of lowered connectivity. Such initiatives include fee waivers for author processing charges to enable LMIC scientists to publish articles (and the corresponding datasets) in Open Access journals. Moreover, research consortia (such as the MalariaGen network) and journals have started to allow LMIC scientists extended periods of time in which to deposit their data post-publication (so as to ensure that they are able to fully exploit the datasets for publication purposes).

Policies such as the MalariaGen moratoria have been particularly effective since implementation (de Vries et al., 2011), as it was designed in response to concerns highlighted by the LMIC scientists within the research network. The success of such initiatives highlights the importance of better understanding how data sharing (dis)incentives are framed by LMIC science communities. Indeed, as further data sharing support initiatives are developed for roll-out in LMICs, it becomes evident that far more information is needed on how data sharing in perceived in LMICs. Do characteristics of their research environments pose unique disincentives that would discourage them from data sharing? Are the data sharing incentives discussed above sufficient to motivate sharing?

## Methods

What the qualitative studies on data sharing in LMICs have shown is how closely intertwined scientists’ opinions on openness, sharing and research are with the environment in which they are working (Bezuidenhout, Kelly, et al., 2017; Bezuidenhout, Rappert, Leonelli, & Kelly, 2016). In order to expand on these studies, a quantitative survey was prepared for life scientists in LMICs on data sharing that particularly interrogated elements of their research environment. The survey asked respondents about their daily data sharing practices, while also interrogating physical, social and regulatory resource provision within their specific research institution. A copy of the survey may be found at the link noted in the footnote below.^6^

This survey was disseminated to life scientists who were members of the NEPAD (the New Partnership for Africa’s Development)-Southern African Network for the Biosciences (NEPAD-SANBio) network. NEPAD-SANBio is a platform for sharing research, development and innovation and was established in 2005 under NEPAD. The NEPAD-SANBio network covers 13 countries: Angola, Botswana, Malawi, Mauritius, Mozambique, Namibia, Lesotho, Swaziland, Seychelles, Madagascar, South Africa, Zambia and Zimbabwe. The network consists of both institutions and individual scientists.

This network offered a valuable resource for this project as a sample population as it provided us with access to a population of scientists who were not only regularly online but also potentially – through their membership to the network – interested or engaged in data sharing activities. Consequentially, the network provided a sample population who represented African researchers who would be more likely to engage in data sharing than many of their colleagues.

NEPAD-SANBio operates as a distributed (“hub and spoke”) network, with contact points in each of the 13 countries. A link to the online survey was disseminated via the NEPAD-SANBio Secretariat in August 2016 to each national contact point who were asked to disseminate it to their national member group. As communication between the contact point and national membership body can vary, it is thus difficult to estimate the total number of individual members who received the survey link. Nonetheless, the final dataset had representation from each country within the SANBio network.

The survey was completed online via the SurveyMonkey online survey platform. The homepage to the survey contained a full description of the survey, the uses of the data gathered, and issues of data storage and privacy. Completion of the survey was taken to indicate consent for participation and data re-use, as detailed on the homepage. The data collected during the survey were fully anonymized and individual contributors were not identifiable. All datasets collected during the survey were stored securely on a password-protected computer and only accessible to the researchers named on this project and securely backed up on a password-protected server. The data from this survey will be stored for a maximum of two years after the completion of the project.

In total, 100 responses were collected via the online platform, and all 13 countries covered by the NEPAD-SANBio network were represented in the sample. While certain countries (such as South Africa) were more highly represented than others (such as Angola), the higher number of research institutions within these countries made this unsurprising. The low number of responses from certain countries made country-specific analyses impossible, so the entire dataset was analysed together. The salient demographic information about the survey population is listed in Table 1.

**Table 1.** Demographic data collected.

Country	Number of responses	Position	Number of responses	Funding source	Number of responses
South Africa Zimbabwe Namibia Lesotho Botswana Malawi Zambia Swaziland Mauritius Mozambique Angola Seychelles Madagascar	31 23 9 7 7 6 5 4 3 2 1 1 1	Professor Lecturer/researcher Postdoctoral Researcher Postgraduate student Place of work University College Government research Independent research Facility Industry	14 57 3 26 Number of responses 60 0 27 10 3	International grant National grant Private sector Internal funding No funding Number of published, peer-reviewed papers over 5 years None 1–3 3–5 Over 5	27 45 2 6 20 Number of responses 26 42 8 24

The responses are presented thematically below, drawing on key incentives and disincentives identified from the Wiley survey (Ferguson, 2014) that was rolled out in the U.S.A., U.K., Japan, China, Brazil Australia and Germany. In this survey, respondents identified standard practice within research communities (57%) and increased impact and visibility of research (55%) as top incentives for data sharing. The first section of the results/discussion, therefore, offers an analysis that looks at the incentive of sharing data to increase research visibility based on the data from the survey.

The lowest disincentive for sharing data identified in the Wiley survey was a lack of funding (11%). The second section of the results/discussion analyses how the lack of funding acted as a major disincentive for data sharing amongst the survey respondents. The final section considers a disincentive that is not mentioned in the Wiley survey, namely issues of connectivity. All three sections are presented with results and discussion, as well as some key questions that need to be considered in future LMIC data sharing initiatives.

## Increased impact and visibility of research

The opportunity to increase the impact and visibility of one’s research through data sharing is commonly cited as an incentive. Indeed, in the Wiley survey discussed above, 55% of respondents identified this as a key benefit of data sharing. Nevertheless, properly unpacking this incentive requires that a wide range of other issues are considered, such as the pathways and platforms for sharing that truly increase visibility, the skills and support to use these pathways, and the *in situ* evidence of the benefits of engaging in such sharing. Are such elements in place within LMICs so as to truly translate this hypothetical incentive into a true motivator?

### Results

In a similar fashion to the results of the Wiley survey, our respondents recognised the value of sharing data as a means of increasing research visibility. Interestingly, however, far more respondents were interested in using data sharing as a means of establishing future *personal* connections than simply as a means of improving research visibility (Table 2).

**Table 2.** Responses to the question: I believe the biggest benefit of sharing my own data is … (select one option).

The biggest benefit of sharing my own data is	Percentage of responses
It brings networking and collaboration opportunities	47
It contributes to the advancement of science	41
It contributes to the visibility of my research	11
I don’t believe there is a benefit	1

Respondents were also asked to select from a variety of potential data sharing activities that they used in their daily research. While few engaged with “altmetric” sharing platforms such as Figshare, the vast majority of respondents published, emailed colleagues and maintained professional networking profiles on ResearchGate (see Table 3). Perhaps from lack of institutional support, however, only 39% of respondents maintained a personal web page linked to their institution or project.

**Table 3.** Responses to question: Now could you let us know what sort of online activities you are normally engaged with. Please check *all* the appropriate answers to the statement: I regularly share data and publications via …

Online activity	Percentage of respondents
Peer-reviewed publication	80
Altmetric websites such as Figshare	16
Email with colleagues	80
Professional networking sites such as ResearchGate	73
Institutional repositories	53
Online databases	58
Personal or project web pages	39

The preference towards *personal* connections with potential data recipients was further evident in the responses to the questions “with whom would you share data, and when”? While over two-thirds of respondents were comfortable to share data after publication, the situation was very different for sharing pre-publication data. While 60% of respondents said that they were comfortable sharing pre-publication data with people that they knew, only 13% would consider sharing pre-publication data with people they did not know (Table 4).

**Table 4.** Responses to questions: I am comfortable sharing my data with people I know/do not know … Please tick *all* appropriate responses.

When to share data	With people that I know (percentage of responses)	With people that I do not know (percentage of responses)
Pre-publication	60	13
Post-publication	74	65
Only through publication	44	55

Respondents were then asked to rank their concerns about sharing data. As is evident in Table 5, the most pressing concern was having other researchers take results (34%).

**Table 5.** Responses to the question: my biggest concern about sharing my own data is … (select one option).

Concerns about sharing data	Percentage of respondents
Having other researchers take my results	34
Having my data mis-interpreted or mis-attributed	29
Missing out on opportunities to maximize intellectual property	23
Losing out on opportunities to maximize my publications	14

### Discussion and key questions

The possibility of “being scooped” is regularly cited as a disincentive against data sharing, regardless of where the scientists are located. This is particularly true of unpublished data, where there is always the chance that someone else will be able to analyse and publish from the data and thus gain credit. Current discussions about removing the disincentive of “scooping” have mainly focused on strengthening the systems that facilitate better credit attribution (such as Creative Commons) and transparency.

It is questionable whether better credit attribution systems will similarly serve to remove the disincentive of “scooping” in LMICs. In qualitative research carried out by Bezuidenhout, Kelly, et al. (2017, p. 11) succinctly elaborated on the fears of being “scooped”, saying: “because it takes us so long to complete our research, other people have a lot of opportunities to steal our data. We must keep it secure until we publish”. This idea of keeping data secure was further reiterated in statements such as: “[e]ven when you’re hiding your data, anyone can run away with it”.

Such statements shed insight into the marked difference between the willingness to share data with known or unknown people, as described in Table 4. Because of the longer time it takes to “do research” in LMICs, it would seem that scientists want to “keep an eye” on their data until they are ready to publish. Sharing – particularly pre-publication – is thus mediated in terms of trust and personal connections. Enhanced credit attribution is unlikely to fully address such concerns.

Nonetheless, a considerable amount of online visibility hinges on sharing pre-publication data. This could be as pre-publication journal article drafts, research project descriptions, research updates, conference proceedings and directly upon informal request. Unless researchers publish frequently and regularly their decisions not to release pre-publication data can be highly detrimental. Such observations were particularly important for our cohort, in which as only 25% of respondents had published more than 3 papers in the last 5 years. Without the pre-publication release of data, research taking this long time to be published makes it likely that a considerable amount of the data produced in LMIC research is regularly overlooked. Data from LMIC research endeavours, instead of being effectively disseminated and re-used, is languishing in drawers, hard-drives or on Dropbox for many years due to issues of trust and personal connection.

These observations indicate a seemingly intractable bind. LMIC research – and researchers – are often overlooked because of their lack of online visibility. Particularly, the absence of pre-publication data and the slow rate of academic publications diminish the impact that LMIC research has in the global research community. Nonetheless, it is precisely because of the slow rate of publication that LMIC scientists are hesitant to share pre-publication data with anyone with whom they do not have a personal connection. Interestingly, the absence of engagement in altmetric pathways – that many would assume is a form of connection – suggests that what constitutes “personal” for LMIC scientists requires considerable further interrogation. This raises a number of key questions detailed below.

1. How can Open Data engagement strategies be structured for use in LMICs that take into account the current data practices, and address issues of scooping in ways that reflect the low-resourced research context that most researchers will be working in?
2. What elements of the “personal connection” act as key motivators for data sharing – is it accountability, trust, direct access to retribution or something different?
3. What constitutes a personal connection for LMIC scientists – it is a physical meeting, an online interaction or an affiliation through an acquaintance. Do network memberships constitute a “mediated intermediary” between people you have met and the “other”?
4. Can research networks such as NEPAD-SANBio and professional organizations initiate discussion – and provide support – for dissemination plans that address both increased visibility and the need for academic publications?

## Funding for data sharing/data sharing as a funding requirement

The issues surrounding funding and data sharing are two-fold. Data sharing is recognized to require financial, time and human resources, as well as infrastructures that support these activities. Such resources are usually provided by institutions and funding bodies. In return, an increasing number of funding bodies, institutions and national governments have specific data sharing expectations in return for research funds. While still a complicated set of issues in HICs, evidence of progress in this area is evident from the Wiley survey, where the lowest disincentive for sharing data survey was a lack of funding (11%) while data sharing as a funding requirement served as a noted incentive (23%). Whether the same nexus of funding incentives/disincentives existed in LMICs, however, cannot be assumed.

### Results

Sixty per cent of the survey respondents felt that they lacked money to conduct research (Table 6). However, it is important that this is not taken as an absolute lack of funding. As detailed in Table 1, the responses showed a wide range of different funding sources for research. These included international grants (27%), national/institutional grants (45%) and private sector funding (2%).^7^ In total, 74% of respondents said that they received some form of funding – in contrast to the 25% who had no outside funding or no funding at all. While the size of the grants, of course, may vary considerably, such responses highlight that many researchers have access to some form of research funding, and thus some interactions with funding bodies.

**Table 6.** Answer to question: on a scale from strongly disagree (1) to strongly agree (5), please rate the following statements … (in percentage of respondents).

	1	2	3	4	5	N/A
I lack money to conduct research	5	16	14	21	39	5
I do not have the flexibility to use research money to address core issues in my laboratory	6	18	20	25	21	10
I lack funds to maintain and upgrade my laboratory environment	5	6	12	20	45	12
I lack the ability to spend the money I have in ways that are most necessary for my research	12	32	17	17	15	7
Data sharing is not part of promotion criteria	3	23	21	31	17	5

Despite respondents reporting some level of financial support for their research, it was also salient to note additional financial challenges that they faced in daily research. These included perceptions that they were not able to use the available funds to maintain and upgrade their laboratory environments (65% agree/strongly agree), or have the ability to address core issues within their laboratories (46% agree/strongly agree). This undoubtedly impacted on their ability to create and maintain environments permissive towards data sharing – something that was compounded by data sharing rarely being a criterion for promotion.

### Discussion and key questions

Funding for research is always a thorny issue amongst researchers, and most would suggest that they lack funding to do *all* the research they want. When considering funding landscapes, it is reasonable to assume that researchers in LMICs struggle more than most. From a binary perspective, the answer seems relatively straightforward: increase the amount of funding to LMICs and there will not only be more data produced, but likely more data online. The assumption of a chronic shortage of funding – on institutional, national and private levels – in LMIC higher educational institutions has been a highly productive tool for the Open Access community to motivate for journal fee waivers and discounted access bundles for libraries, and it is tempting to think the same true for Open Data. Nonetheless, assuming a shortage of funding is equated with a lack of funding is also deeply problematic.

These results seem to show that responses to contentious issues such as research funding depend on what questions are asked. Regardless of whether the survey respondents received funding for research, the results from Table 6 suggest areas of chronic underfunding that are not only overlooked, but critical to the development of robust data sharing activities. The respondents from this survey felt that they were unable to maintain or upgrade their current research environments with the funds available to them. This suggests that the establishment or perpetuation of data sharing activities that required dedicated resources was extremely difficult for them. Such observations strongly point to the need for the Open Data community to examine the issue of funding for data sharing from a more holistic, systemic perspective than currently occurs.

As evident in the Global North, funding bodies play an important role in making data sharing part of daily research activities. In contrast, the results of this survey suggest that this power has yet to be exploited by many funders operating in LMICs. Nevertheless, recognizing this caveat cannot simply be a case of stricter data sharing requirements. As mentioned above, many funders, collaborations and journals allow LMIC researchers extended time-periods in which to release the data associated with publications. Moreover, even within South Africa – the most productive research nation in Africa – the average researcher produces 0.63 papers a year (which is equivalent to 3 per 5 years), making the benchmark for comparison very low compared to the HIC average. It is therefore apparent that even if stricter data sharing requirements *post-publication* were introduced, this would not stimulate rapid data dissemination in LMICs. There, therefore, needs to be a critical analysis of the role of funding bodies in LMIC data production and release that examines possible alternative ways of incentivizing data sharing that would be beneficial to these science communities.

In doing so, it is important to recognize a key driver of this bind – the well-recognized link between promotion criteria and publication of peer-reviewed journal articles in most African universities (Bezuidenhout, Kelly, et al., 2017). Indeed, within our study cohort, this was no different, with 72% of respondents agreeing that they did not receive support for data sharing and dissemination aside from publishing in peer-reviewed journals. It would thus seem that current promotion criteria in many African universities lock researchers into traditional avenues of dissemination-via-publication, which not only slow down the rate at which data are released from these sites, but also potentially decreases impact. Many publications recognize that judging impact solely by citations is not only potentially misleading (Falagas, Kouranos, Arencibia-Jorge, & Karageorgopoulos, 2008; PLoS Medicine Editors, 2006; Wilhite & Fong, 2012) but also painfully slow (Brody, Harnad, & Carr, 2006), and overlook increasingly important societal and clinical impacts (Lewison, 2002). While such systems remain in place, it is likely that funders will continue to get sub-optimal returns on their investments, and that data will continue to be inefficiently utilized and disseminated.

If the research environments in LMICs are slowing down not only data production, but also data sharing more needs to be done. In particular, there needs to be more engagement with LMIC scientists to identify ways through which to speed up their research – particularly if they are still tied to data sharing through publication (promotion). This raises a number of key questions, detailed below.

1. What is causing such a low level of publication returns on funding investments? Do funding structures need to be revisited to address issues of “expected returns”?
2. How can discussions about research infrastructures become part of funding discussions on data sharing?
3. Can promotion criteria in LMICs be critically examined to include credit for data sharing in a productive manner? Can discussions also be initiated relating to “quality versus quantity” publications?
4. Are there ways in which grants can initiate discussions on sharing that are not solely tied to post-publication sharing?

## Recognizing a “continuum of access”

A key element of discussions about Open Data in Africa has been the existence and perpetuation of a “digital divide” – the perceived absence of ICTs, Internet provision or computer skills necessary for effective online participation (Bezuidenhout, Leonelli, et al., 2017). While issues of access, of course, remain important considerations, recent ethnographic research revealed hidden complexities (Bezuidenhout, Kelly, et al., 2017). Challenges such as the age of the hard- and software being used, the frequency of power-cuts interrupting Internet provision and poor personal Internet connection were all cited as key factors shaping researchers’ ability to work online. The ability to work online is therefore better understood in terms of a “continuum of access” rather than as a binary switch from nothing to online productivity (Bezuidenhout, Kelly, et al., 2017).

Nonetheless, these mundane, daily challenges of effective online activity are often overlooked. This is salient for two different reasons – first, that such issues are rarely featured discussions about Open Data (due to the online/offline focus), and second, because such issues are so much part of many African research environments that even researchers may fail to recognize their key significance. As a result, it is highly likely that when African scientists are asked about ICT challenges in their research environments either the questions will not reflect the challenges that truly influence their daily activities, or they will not recognize their import.

### Results

It is important to recognize the limitations of making use of online survey software such as SurveyMonkey. All the respondents would, of course, have access to the Internet and a computer. Nonetheless, assuming that the ability to get online suggests that there are no problems with online access further exemplifies the limitations of any online/offline binary position, as discussed above.

Table 7 summarizes the participants perceptions of how different infrastructural challenges affected their research. Despite their online connectivity, over half of respondents agreed/strongly agreed that the absence of up-to-date hardware (61%) and software (58%) curtailed their ability to engage online. Similarly, while all respondents had access to the Internet, and the speed of institutional cable Internet (52% agree/strongly agree), institutional wifi (54% agree/strongly agree) were identified as key factors limiting online activities.

**Table 7.** Responses to the question: on a scale from strongly disagree (1) to strongly agree (5), please rate the following statements … (in percentage of respondents).

	1	2	3	4	5	N/A
Power outages challenge my ability to generate data	11	24	14	25	21	5
Power outages challenge my ability to find and re-use data online	12	26	12	33	13	4
I lack up-to-date hardware	9	19	7	25	36	4
I lack up-to-date software	10	19	10	22	36	3
The speed of the cable Internet at my university slows down my online activities	8	19	12	21	31	9
The speed of the wifi at my university slows down my online activities	6	17	13	19	35	10
I do not have good Internet connection at home, which affects my online activities	10	15	10	23	40	2

Moreover, 63% agreed/strongly agreed with the statement: I do not have a good Internet connection at home, which affects my online activities. This correlates with previous qualitative findings (Bezuidenhout et al., 2016; Bezuidenhout, Kelly, et al., 2017) that emphasize the difficulties that many African researchers have in controlling when and where they choose to work online.

### Discussion and key questions

Such results present a complicated picture of the online activities of the respondents. While these survey respondents represented perhaps some of the most “online” of researchers in sub-Saharan Africa (by virtue of being online, being part of NEPAD-SANBio, and being willing to respond to the survey), the ICT challenges they experienced were notable. These data clearly highlight the need for nuanced interpretations of connectivity, and the key issues that disappear in binary interpretations of access.

These results draw attention to the dangers of assuming that just because an LMIC scientist is online that their online activity will be similar to their colleagues in HICs. Contending with daily ICT activities that take longer than in the Global North, being unable to use certain platforms due to software restrictions, and being unable to control when and where one chooses to work all significantly impact on the ability (and enthusiasm) to share data. Without dedicated attention to these issues, it is unlikely that any of the incentives for data sharing that are being developed will gain traction in the Global South. This raises some key questions for the Open Data community that are listed below.

1. How can discussions about “poor ICT access” be differentiated from those of “no access” in data sharing discussions?
2. What can be done to address the “continuum of access” within LMIC research settings?

## The same fears … but different?

If LMICs are to reach the development goals that they are beginning to identify, then science, technology and innovation are key (Marsh, 2016). Commitments from governments, the international science community and key stakeholders, together with the move towards Open Data, can make this happen. Nonetheless, if key elements of working in low-resourced research environments continue to be overlooked, the scope and efficiency of this development may be compromised.

The evidence presented in this paper suggest two key issues that need to be carefully unpacked. First, that the discourse surrounding (dis)incentivizing data sharing cannot be assumed to translate wholesale from the Global North to the Global South. Indeed, many of the concerns surrounding data sharing – while looking markedly similar from the outside – play out significantly differently in low-resourced research environments. This changes the dynamics of individual scientists’ interactions with Open Data discussions and the development of data sharing practices. Continuing to assume that LMIC scientists are motivated by the same set of concerns as their HIC counterparts has the real potential of further marginalizing them from the developing Open Science landscape.

Second, the data show the impact that low-resourced research environments can have on the establishment of robust communities of data sharing in LMICs. Without further empirical research to scope out the extent of this interaction, it is unlikely that evidence-based data sharing policies will be developed to truly initiate an Open Data landscape in the Global South. It is, therefore, an imperative that the Open Data community – scientists, funders and national/international stakeholders – dedicate time and resources to properly understanding the current binds of LMICs and to identifying appropriate solutions.

Despite such cautionary words, however, the interest in data sharing amongst LMIC scientists is ever-growing. Scientists in these regions recognize the incredible potential of an Open Science future, and their enthusiasm for being part of it is something that needs to be capitalized on. It will be by working with these scientists that we will be able to identify the most sensible ways to bring them into such futures.

## Notes

1 Such as *The Alliance for Accelerating Excellence in Science in Africa* (AESA). See aesa.ac.ke/ (12 December 2017).

2 Such as for the NEPAD-SANBio Southern African Network for Biosciences.

3
http://africanopenscience.org.za/wp-content/uploads/2017/07/brief.pdf (12 December 2017).

4
https://wellcome.ac.uk/funding/managing-grant/policy-data-management-and-sharing

5 Priem, Piwowar, and Hemminger (2012) identifies a number of different areas of altmetric activity, including social media like *Twitter* and *Facebook*, online reference managers like *CiteULike*, *Zotero*, and *Mendeley*, collaborative encyclopedias like *Wikipedia*, blogs, both scholarly and general-audience, scholarly social networks, like *ResearchGate* or *Academia.edu*, conference organization sites like *Lanyrd.com.*

6
https://figshare.com/articles/INASP_survey_final_pdf/4818043 (accessed 5 April 2017).

7 This would seem in line with Africa Innovation Outlook 2010 and 2014, which clearly show that the government is the major source of funding for R&D and followed by international grants, in most African countries. Private-sector support for research is quite negligible (AU-NEPAD, 2010; NEPAD, 2014).
